# Impact of Pain on Activities of Daily Living in Older Adults: A Cross-Sectional Analysis of Korean Longitudinal Study of Aging (KLoSA)

**DOI:** 10.3390/geriatrics9030065

**Published:** 2024-05-20

**Authors:** Ambrish Singh, Sreelatha Akkala, Minakshi Nayak, Anirudh Kotlo, Naresh Poondla, Syed Raza, Jim Stankovich, Benny Antony

**Affiliations:** 1 Menzies Institute for Medical Research, University of Tasmania, Hobart 7001, Australia; ambrish.singh@utas.edu.au (A.S.);; 2 School of Public Health, The University of Texas Health Science Center at Houston, Houston, TX 77030, USA; 3 Arthritis Research Canada, Vancouver, BC V5Z 1L7, Canada; anirudhkotlo.19@gmail.com; 4 Department of Biomedical Science, Graduate School of Biomedical Science and Engineering, Hanyang University, Seoul 04763, Republic of Korea; psrnaresh@gmail.com; 5 Department of Neurology, Icahn School of Medicine at Mount Sinai, New York, NY 10029, USA; 6 Independent Consultant HEOR, Mississauga, ON L5R 2C5, Canada; 7 Medical Sciences Precinct, University of Tasmania, Hobart 7001, Australia; jim.stankovich@gmail.com

**Keywords:** pain, elderly, activities of daily living, quality of life, musculoskeletal pain, multisite pain, Korea, aging, older adults

## Abstract

Pain, particularly musculoskeletal (MSK) and multi-site pain, significantly impacts activities of daily living (ADL) in the elderly, leading to a decline in overall quality of life (QoL). This study, comprising 7490 participants, (mean age: 69 ± 10; females: 57%) from the sixth wave of the Korean Longitudinal Study of Aging (KLoSA), aimed to assess the association between self-reported pain and ADL impairment among the elderly population. Notably, 62% of participants reported experiencing pain, with back pain being the most prevalent (36%) and stomachache the least (0.39%). A majority (61%) of individuals reported MSK-related pain. Additionally, 20% reported pain at one site and 0.03% experienced pain at 12 sites. ADL impairment was observed in 376 (5.0%) participants. Compared to those without pain, participants reporting moderate and severe pain had higher odds of ADL impairment [2.31 (95% CI, 1.66–3.21) and 2.98 (95% CI, 1.95–4.53), respectively]. Pain experienced in the shoulder, arm, wrist, back, hip, leg, and ankle had a significant association with ADL impairment, with ORs ranging from 2.66 (95% CI, 1.80–3.93; hip pain) to 1.36 (95% CI 1.07–1.72; back pain). Furthermore, multi-site pain was associated with higher ADL impairment [1–6 sites: OR: 1.49 (95% CI, 1.11–2.01); 7–12 sites: OR: 7.16 (95% CI, 3.60–14.26)]. These findings underscore the importance of addressing MSK and multi-site pain through targeted interventions, potentially enhancing ADL and contributing to an improved QoL among the elderly population.

## 1. Introduction

In recent years, there has been a notable rise in the aging population attributed to demographic transition. Pain is prevalent among the elderly population, with its frequency and occurrence escalating with each passing decade of life [1,2]. Pain is associated with physical discomfort and reduced mobility, impacting patients’ activities of daily living (ADL), quality of life (QoL), and overall health [3,4,5]. Chronic pain impacts between 2% and 40% of the general population, while an estimated 27% to 58% of individuals aged 65 years or older experience this condition worldwide [3,5,6]. Patients who experience musculoskeletal (MSK) pain often have pain at more than one anatomical site at the same time, referred to as multi-site pain. Multi-site pain affects approximately 25% to 43% of the elderly population [7,8] and is reported to have a significant impact on QoL, healthcare resource utilization (HCRU), and mental health compared to chronic single-site pain [9].

In 2017, around one billion people worldwide were aged 60 years or above. This figure is expected to more than double by 2050 [10]. Older age is associated with impairment in basic ADL including walking, eating, dressing, using the toilet, maintaining continence, and personal hygiene [11,12]. Among the elderly population, the most frequently affected sites for chronic MSK pain are the back, hip, and knees, whereas younger adults commonly report such pain in the lower back, neck, shoulders, and knees [13]. The English Longitudinal Study of Aging (ELSA) has reported a significant association between multi-site pain and limited ADL among individuals aged 50 years and older [13]. Similar results were observed in a population-based study conducted in Taiwan among older adults (n = 2680), where both pain severity and the number of pain sites were significantly associated with functional limitations [14]. Given the substantial burden associated with multi-site pain compared to single-site pain, researchers argue for recognizing multi-site pain as a distinct geriatric syndrome akin to other established geriatric syndromes [8,15]. Multi-site pain has also been associated with late-life depression, [16] the psychosocial health of older women, increased medication use [17], and sleep disturbances [18]. Considering the substantial burden on day-to-day activities, the impact of multi-site pain on ADL among the elderly population needs further exploration.

South Korea has one of the most rapidly aging populations in the world. The impacts of the intensity, type, and site of pain (including multi-site pain and MSK pain) on ADL have not been explored in great depth in this population [19]. While South Korea’s demographic transition towards an older population is one of the fastest worldwide, the aging and its associated challenges such as chronic pain, are global issues. Chronic pain, particularly multi-site pain, presents a significant barrier to ADL, QoL, and overall health across different populations.

The present study uses data from the Korean Longitudinal Study of Aging (KLoSA) to explore the impact of pain on ADL in the South Korean elderly population [20]. By doing so, our study aims to contribute to the broader understanding of the effects of chronic pain in the elderly population.

## 2. Materials and Methods

### 2.1. Design and Participants

We obtained de-identified data from the sixth wave of the KLoSA, encompassing South Korean community-dwelling adults aged 50 years or older. Designed by the Korea Labor Institute, the KLoSA study aims to gather data related to conditions and behavior patterns of older individuals to inform the development of effective socio-economic policies addressing emerging issues associated with the elderly population [21,22]. Initiated in 2006, the KLoSA study is conducted biennially, utilizing a multi-stage stratified sampling approach to ensure that study participants are representative of the South Korean population [22]. The initial wave of the study included 10,254 individuals aged 45 years or older, randomly selected from various provinces across South Korea, achieving an overall response rate of approximately 80.7%. For this study, we utilized data from the sixth wave of the KLoSA, comprising participants aged 50 years or older at the time of the study, totaling 3189 men and 4301 women. The study design involved a cross-sectional analysis of data collected from the participants (aged 50 years or older). The reason for this age cut-off is to focus on the elderly population, in accordance with the study’s main objective.

### 2.2. Measurements

The primary variables of interest were pain status, pain location, pain severity, and ADL. 

The KLoSA survey collected information on pain across 13 areas of the body using a questionnaire [23]. Pain severity was measured on a numerical rating scale (NRS) ranging from 0 (no pain) to 10 (worst pain). MSK pain was defined as pain originating in specific anatomical areas including the shoulders, arms, wrists, fingers, waist, hips, legs, knees, ankles, and toes, while other sites included headache, chest pain, and stomachache. ADL impairment was evaluated using the Katz index of independence, with participants rating their ability to perform six key ADL tasks (dressing, bathing, eating, washing the face, getting out of bed, and using the toilet) independently. Participants reported the level of assistance required for each, using a 3-point scale (1 = no assistance, 2 = partial assistance, 3 = total assistance). A binary ADL variable was created, indicating any difficulty with at least one ADL. Participants were asked to rate their ability to perform each ADL independently on a 3-point scale (0 = no difficulty, 1 = some difficulty, and 2 = unable to do). The scores for all six activities were summed to give an overall ADL score, with higher scores indicating greater impairment in ADLs. 

Demographic characteristics such as age, sex, body mass index (BMI), and physicians-diagnosed co-morbidities, including heart disease, stroke, hypertension, cancer, lung disease, diabetes, arthritis, liver disease, digestive disease, and psychological problems, were collected in the study. 

### 2.3. Ethical Considerations

The KLoSA study has been approved by the Institutional Review Board (IRB) of the Korea Centers for Disease Control and Prevention [24]. Furthermore, since the KLoSA database is publicly released for scientific purposes, ethical approval was not required for this study.

### 2.4. Statistical Analysis

The sociodemographic characteristics of the participants are summarized using means with standard deviations (SDs) for continuous variables and percentages with frequency counts for categorical variables. Multivariable logistic regression, adjusted for age sex and BMI, was performed to estimate the association between self-reported pain (based on pain site and severity) and ADL, and corresponding odds ratios (OR) and 95% confidence intervals (95% CI) were reported. All statistical analyses were performed using STATA software, version 16.1 (Stata Corp, College Station, TX, USA).

## 3. Results

The study included 7490 participants aged 50 years and above, with their demographic and clinical attributes presented in Table 1. The mean age of the participants was 69 years, with the majority of the participants (84%) between 50 and 80 years of age. A quarter of the participants (24%) were classified as obese (BMI > 25 kg/m^2^). Notably, hypertension (42%) and arthritis (25%) were the most prevalent comorbidities within this elderly group (Table 1).

Table 2 presents the frequency of site-specific pain. The majority of participants experiencing pain reported mild intensity, ranging from 40% (ankle pain) to 72% (shoulder pain). Moderate pain prevalence ranged from 24% (shoulder pain) to 49% (ankle pain) of participants, while severe pain was reported by a smaller proportion (3% for arm pain and 12% for hip pain). Among participants reporting any pain, a higher percentage reported severe pain in the hip (12.1%), toe (11.9%), ankle (10.8%), and knee (9.1%) compared to other sites. A similar trend was observed for participants reporting moderate pain. Back pain was the most commonly reported (36%), while stomachache was the least prevalent (0.39%) (Table 2).

Overall, a quarter of respondents reported moderate to severe pain, with MSK pain being the most prevalent (61%). While 21% of participants experienced pain in only one site, only 0.03% experienced pain in 12 sites (Table 3).

### 3.1. Basic Activities of Daily Living

Among participants who required total assistance (n = 123), the largest number needed assistance with bathing (N = 112), followed by those who required assistance with getting out of bed (n = 86) (Table 4).

### 3.2. Association of Pain with ADL

Table 5 presents the results of the logistic regression analysis for the association between pain severity and ADL impairments. Participants with moderate or severe pain had a significantly higher ADL impairment compared to those without pain. The OR for ADL impairment in participants with moderate pain was 2.31 (95% CI: 1.66–3.21), and for severe pain it was 2.98 (95% CI: 1.95–4.53). Furthermore, severe pain in hip, back, ankle, wrist, leg, and arm was significantly associated with ADL impairment. Similarly, moderate pain in the shoulder, ankle, knee, leg, hip, back, chest, finger, wrist, and arm, along with moderate headache, were significantly associated with ADL impairment. Overall, pain in the shoulder, arm, wrist, back, hip, leg, and ankle had a significant association with ADL impairment, with ORs ranging from 1.36 for back pain (95% CI 1.07–1.72) to 2.66 for hip pain (95% CI, 1.80–3.93). Multisite pain was also associated with a greater degree of ADL impairment [1–6 sites: OR (95% CI): 1.49 (1.11–2.01); 7–12 sites: OR (95% CI): 7.16 (3.60–14.26)].

## 4. Discussion

To the best of our knowledge, this study is the first to examine associations between multi-site pain and ADL among the elderly population in South Korea. Consistent with existing research, we observed a high prevalence of self-reported MSK pain (61%) among the elderly population. Notably, ADL impairment significantly correlated with pain severity, and individuals with multi-site pain had a higher ADL impairment. 

Consistent with our findings, earlier studies from Korea have reported similar prevalence of chronic pain (65%) among the elderly [25]. Over a quarter of respondents in our study reported moderate or severe pain at various sites, including the ankle and shoulder (40–72%). These findings are consistent with a South Korean cohort study which showed an increase in lower extremity (LE) MSK pain prevalence with age, peaking at the age of 80 or older (unadjusted OR = 2.649). Individuals aged 75–79 had a higher prevalence of upper extremity (UE) pain and low back pain compared to those aged 65–69 [26]. Additionally, data from the Japan Gerontological Evaluation Study (JAGES) analyzing data from over 12,000 elderly people (>65 years) found that the top three sites for chronic MSK pain prevalence were shoulder (15%), lower back (14%), and knee (12%) [27].

The rapid growth of the elderly population globally necessitates further research on MSK pain. Recent studies reveal a high prevalence of chronic pain among the elderly, ranging from 39% to 70% worldwide [28,29,30,31]. In China, studies have showed a chronic pain prevalence of around 58% among the elderly, and in Japan, the prevalence is notably high, with rates of 62% reported among individuals aged 60–69 years and 72% among those aged 70 years and older [27,32]. Similarly, a study conducted in a single Japanese city reported a chronic pain prevalence of over 50% among people aged between 60 and 89 [27]. Similar findings have been observed among elderly people in the India, with reported prevalence of chronic pain ranging from 30 to 72% [33,34].

Our study findings add to the existing literature on MSK pain in the elderly population, highlighting its significant impact on QoL and functional ability. MSK pain is a leading contributor to disability worldwide, with recent data suggesting that low back pain is the primary cause of disability in 160 countries [35,36]. Our results showed that 61% of the elderly population (>50 years) reported MSK-related pain, with back pain being the most commonly reported (36%). In comparison, a previous study of the Korean elderly population reported a higher crude prevalence of MSK pain for UE pain (61.5%), low back pain (72.0%), and LE pain (43.9%) [26]. These findings highlight the importance of addressing MSK pain in the elderly population, given its substantial impact on QoL and functional abilities. In our study, 21% of participants reported pain in one site, and 41% reported pain in two or more sites (referred to as multisite pain). Previous studies among the elderly have reported prevalence of multisite pain ranging from 41% to 75% [15,37,38]. Compared to single-site pain, our findings suggest that multi-site pain presents more complex challenges to overall health affecting ADL. A cross-sectional study from the PainS65+ cohort, consisting of participants residing in the southeastern Sweden, showed that approximately 13% of the respondents reported pain at one pain site, and nearly 39% reported pain at two or more sites. The most common anatomical painful sites were the LE, UE, the gluteal area, and the low back (72%, 45%, 13%, and 14%, respectively) [39]. Another population-based cohort study from the US showed that, among those with chronic pain at any particular MSK site, between 75% and 90% reported pain in one or more other sites [8]. This comparison highlights our study’s distinct emphasis with the broader findings from a US cohort study that emphasizes the widespread nature of chronic multi-site pain. This also suggests that interventions targeting multi-site pain could yield significant benefits for the elderly population worldwide. 

Turning to pain severity, around 25% of participants reported moderate and severe pain, with severe pain having a prevalence of 15%. Most patients who experienced moderate to severe pain had MSK pain. These findings are consistent with previous studies reporting a high prevalence of moderate to severe pain in the general population. For instance, Shin et al. (2016) conducted a study in Korean adults (mean age: 50 years), and reported a prevalence of mild and severe pain of 29.8%, with severe pain having a prevalence of 1.7% [40]. Our study observed a higher prevalence of severe pain, possibly because of the participants’ higher mean age (69 years). Additionally, the definition of multi-site pain may differ across studies, potentially contributing to observed variations in pain severity. Factors influencing this variation include the type of pain intensity scale used and the system used to classify pain.

In our study, ADL impairment was apparent among participants experiencing moderate and severe pain. This is consistent with previous research highlighting the association between chronic pain and ADL impairment in older adults. ADL impairment could further lead to mobility limitations, increased functional dependence, and a higher risk of mental health problems [26,41]. Studies have shown that older adults with multi-site pain experience a higher risk of mobility difficulties (e.g., walking and climbing stairs) and impairments in both basic ADL and instrumental ADL (e.g., shopping, preparing meals) [42]. A population-based survey in Europe among people aged ≥65 years with MSK pain showed significant ADL impairment among those with MSK pain compared to those without the disease [41]. 

Studies conducted in other countries have also demonstrated a significant correlation between pain and ADL impairment. A US-based study reported significant correlation between pain and ADL impairment. Significant correlations were found between five pain rating scales for various ADLs (r = 0.57–0.99) [43]. In a Chinese study, conducted in elderly population, a greater ability to perform ADL was negatively associated with total pain scores (*p* < 0.001) [14]. A recent Japanese longitudinal study reported that, over 3 years, the odds of ADL decline were 50% higher among those with chronic pain than among those without [44]. In line with these findings, our study provides valuable insights into the prevalence of multi-site pain and its association with ADL impairment among older adults. Taken together, these results highlight the need for effective pain management strategies to improve the QoL and functional ability of older adults with pain-related ADL impairments.

A key strength of our study is its generalizability, due to the large sample size and the use of a nationally representative sample from South Korea (KLoSA). However, we acknowledge the potential for recall bias because the data are self-reported. Self-reported ADL impairment may be subject to under- or over-reporting, as participants may not recall their functional status accurately. Additionally, the cross-sectional design limits causal inference, as associations between pain and ADL impairment might be influenced by other factors. It is important to note that these limitations are inherent to cross-sectional studies. Although we adjusted for potential confounders (such as age, sex, and BMI), other unmeasured factors may also contribute to ADL impairment. We acknowledge that our study relied solely on self-reported data collected through questionnaires from the KLoSA, limiting the inclusion of objective assessments such as goniometry, algometry, and gait analysis. While subjective data provide valuable insights into a participant’s experiences of pain and functional impairment, the absence of objective measures may limit the comprehensiveness of our findings. Future research could benefit from incorporating objective assessments to complement self-reported data. This would provide a more comprehensive understanding of the relationship between pain, physical parameters, and ADL among the elderly population.

Furthermore, in order to gain a better understanding of the relationship between multi-site pain and its impact on ADL impairment, future research should delve into the key components and central mechanisms underlying chronic pain. It is critical to differentiate multi-site pain, including MSK pain, from other chronic conditions by clear definitions and measures. Additionally, further investigations into the neural, peripheral, and centrally mediated mechanisms of multi-site pain are required. At the clinical level, healthcare providers should recognize the functional implications of multi-site pain in older adults, and the unique underlying pain mechanisms that significantly impact their physical and psychosocial health. Utilizing multi-site pain measures and pain rating scales is essential when evaluating older adults at potential risk of chronic pain. There is a need for uniform criteria and definitions of multi-site pain. A multidimensional approach is critical to identifying effective interventions that enable personalized treatment strategies to reduce pain and improve QoL for the aging population.

## 5. Conclusions

In conclusion, our findings demonstrate a significant correlation between multi-site pain (specifically MSK pain), pain severity, and ADL impairment among older adults. The prevalence of MSK pain was notably high, with a substantial impact on ADL. The results of our study contribute to a better understanding of chronic pain among the elderly population in South Korea, highlighting the need for targeted interventions to address the unique challenges faced by this group. By identifying specific pain types that significantly affect ADL, such as MSK pain and multi-site pain, our study provides valuable insights for developing targeted interventions to improve QoL for elderly individuals experiencing chronic pain. Furthermore, considering the global demographic shift towards an aging population, our findings have broad relevance beyond South Korea. As countries worldwide progress further through similar demographic transitions, understanding the impact of chronic pain among the elderly becomes critical to inform healthcare policies and interventions. By recognizing the significance of multi-site pain and its association with ADL impairment, our study contributes to the growing body of literature aimed at addressing the complex needs of the aging population globally.

## Figures and Tables

**Table 1:** Socio-demographic and clinical characteristics of the participants.

Socio-Demographic Characteristics	N (%)	Male	Female
Sample, n (%)	7301 (100)	3189 (42.58)	4301 (57.42)
Mean Age years (SD)	69.09 ± 10.33	68.65 ± 9.86	69.42 ± 10.66
Age 50–60, n (%)	1999 (26.69)	862 (27.03)	1137 (26.44)
Age 61–70, n (%)	2229 (29.76)	993 (31.14)	1236 (28.74)
Age 71–80, n (%)	2075 (27.70)	889 (27.88)	1186 (27.57)
Age 81 and over, n (%)	1187 (15.85)	445 (13.95)	742 (17.25)
Mean BMI (SD)	23.27 ± 2.72	23.22 (2.49)	23.32 (2.88)
Underweight [BMI < 18.5 kg/m^2^] n (%)	273 (3.82)	112 (3.66)	161 (3.95)
Normal [BMI 18.5–22.9 kg/m^2^] n (%)	3128 (43.78)	1304 (42.56)	1824 (44.69)
Overweight [BMI 23–24.9 kg/m^2^] n (%)	1996 (27.94)	976 (31.85)	1020 (24.99)
Class I obesity [BMI 25–29.9 kg/m^2^] n (%)	1658 (23.21)	650 (21.21)	1008 (24.70)
Class II obesity [BMI 30–34.9 kg/m^2^] n (%)	86 (1.20)	22 (0.72)	64 (1.57)
Class III obesity [BMI ≥ 35 kg/m^2^] n (%)	4 (0.06)	0 (0)	4 (0.10)
Doctor diagnosed co-morbidities, n (%)			
Heart problem	673 (8.99)	276 (8.65)	397 (9.23)
Stroke	456 (6.09)	227 (7.12)	229 (5.33)
Hypertension	3139 (41.91)	1245 (39.04)	1894 (44.04)
Cancer	456 (6.09)	182 (5.71)	274 (6.37)
Lung disease	227 (3.03)	182 (5.71)	274 (6.37)
Diabetes	1396 (18.64)	605 (18.97)	791 (18.39)
Arthritis	1853 (24.74)	341 (10.69)	1512 (35.15)
Liver disease	196 (2.62)	107 (3.36)	89 (2.07)
Digestive disease	235 (3.14)	78 (2.45)	157 (3.65)
Psychological problem	347 (4.63)	111 (3.48)	236 (5.49)

BMI: Body mass index, SD: Standard deviation; Obesity defined based on BMI as per Korean Society for the Study of Obesity guideline [Journal of Obesity & Metabolic Syndrome 2023, 32, 121–129]; Self-reported pain.

**Table 2:** Pain sites and severity of pain.

Self-Reported Pain	N (%)				
	No Pain	Any Pain	Mild	Moderate	Severe
Headache	7283 (97)	207 (2.76)	142 (68.60)	52 (25.12)	13 (6.28)
Shoulder pain	6397 (85)	1093 (14.59)	790 (72.28)	264 (24.15)	39 (3.57)
Arm pain	6936 (91)	554 (7.40)	379 (68.41)	159 (28.70)	16 (2.89)
Wrist pain	7118 (95)	372 (4.97)	242 (65.05)	110 (29.57)	20 (5.38)
Finger pain	7241 (97)	249 (3.32)	146 (58.63)	89 (35.74)	14 (5.62)
Chest pain	7437 (99)	53 (0.71)	33 (62.26)	17 (32.08)	3 (5.66)
Stomachache	7461 (99)	29 (0.39)	20 (68.97)	8 (27.59)	1 (3.45)
Back pain	4796 (64)	2694 (35.97)	1620 (60.13)	878 (32.59)	196 (7.28)
Hip pain	7226 (96)	264 (3.52)	123 (46.59)	109 (41.29)	32 (12.12)
Leg pain	5305 (71)	2185 (29.17)	1257 (57.53)	744 (34.05)	184 (8.42)
Knee pain	4966 (66)	2524 (33.70)	1372 (54.36)	921 (36.49)	231 (9.15)
Ankle pain	7277 (97)	213 (2.84)	85 (39.91)	105 (49.30)	23 (10.80)
Toe pain	7423 (99)	67 (0.89)	39 (58.21)	20 (29.85)	8 (11.94)

**Table 3:** Percentage of participants reporting pain classified by pain type.

Pain Type	Yes, n (%)
Musculoskeletal pain	4569 (61.00)
Other pain	272 (3.63)
**Number of joints**	
No pain	2869 (38.3)
One site	1551 (20.71)
Two sites	1503 (20.07)
Three sites	915 (12.22)
Four sites	346 (4.62)
Five sites	163 (2.18)
Six sites	74 (0.99)
Seven sites	32 (0.43)
Eight sites	15 (0.2)
Nine sites	12 (0.16)
Ten sites	5 (0.07)
Eleven sites	3 (0.04)
Twelve sites	2 (0.03)

Musculoskeletal pain: shoulder pain, arm pain, wrist pain, finger pain, back pain, hip pain, leg pain, knee pain, ankle pain, or toe pain; Other pain: headache, chest pain, or stomachache.

**Table 4:** Level of assistance required for basic activities of daily living.

Basic Activities of Daily Living	No Assistance Needed, n (%)	Partial Assistance Needed, n (%)	Total Assistance Needed, n (%)
Dressing	7279 (97.18)	140 (1.87)	71 (0.95)
Washing the face	7274 (97.12)	140 (1.87)	76 (1.01)
Bathing	7153 (95.50)	225 (3.00)	112 (1.50)
Eating	7321 (97.74)	97 (1.30)	72 (0.96)
Getting out of bed	7274 (97.12)	130 (1.74)	86 (1.15)
Using toilet	7305 (97.53)	111 (1.48)	74 (0.99)
Any ADL	7114 (94.98)	253 (3.38)	123 (1.64)

ADL items were coded using a 3-point rating scale: 1 (no assistance needed), 2 (partial assistance needed), and 3 (total assistance needed).

**Table 5:** Association between intensity of pain at different sites and ADL.

Self-Reported Pain, n	ADL, n (Row %)		OR (CI)
	Yes	No	
**Any pain (yes/no):** 4621	308 (6.67)	4313 (93.33)	**1.56 (1.16–2.10)**
No pain: 2869	68 (2.37)	2801 (97.63)	Ref
Mild pain: 2663	89 (3.34)	2574 (96.66)	0.89 (0.63–1.27)
Moderate: 1560	159 (10.19)	1401 (89.81)	**2.31 (1.66–3.21)**
Severe: 398	60 (15.08)	338 (84.92)	**2.98 (1.95–4.53)**
**Headache (yes/no):** 207	22 (10.63)	185 (89.37)	1.64 (0.95–2.87)
No headache: 7283	354 (4.86)	6929 (95.14)	Ref
Mild headache: 142	11 (7.75)	131 (92.25)	1.05 (0.48–2.30)
Moderate headache: 52	8 (15.38)	44 (84.62)	**3.32 (1.46–7.58)**
Severe headache: 13	3 (23.08)	10 (76.92)	1.57 (0.26–9.30)
**Shoulder pain (yes/no):** 1093	72 (6.59)	1021 (93.41)	**1.43 (1.05–1.93)**
No Shoulder pain: 6397	304 (4.75)	6093 (95.25)	Ref
Mild Shoulder pain: 790	42 (5.32)	748 (94.68)	1.22 (0.85–1.77)
Moderate Shoulder pain: 264	26 (9.85)	238 (90.15)	**1.95 (1.19–3.20)**
Severe Shoulder pain: 39	4 (10.26)	35 (89.74)	1.59 (0.49–5.12)
**Arm pain:** 554	66 (11.91)	488 (88.09)	**2.42 (1.75–3.33)**
No Arm pain: 6936	310 (4.47)	6626 (95.53)	Ref
Mild Arm pain: 379	35 (9.23)	344 (90.77)	**1.88 (1.25–2.84)**
Moderate Arm pain: 159	28 (17.61)	131 (82.39)	**3.51 (2.14–5.77)**
Severe Arm pain: 16	3 (18.75)	13 (81.25)	**3.97 (1.06–14.82)**
**Wrist pain (yes/no):** 372	42 (11.29)	330 (88.71)	**2.17 (1.46–3.24)**
No Wrist pain: 7118	334 (4.69)	6784 (95.31)	Ref
Mild Wrist pain: 242	19 (7.85)	223 (92.15)	1.52 (0.88–2.61)
Moderate Wrist pain: 110	19 (17.27)	91 (82.73)	**3.35 (1.82–6.15)**
Severe Wrist pain: 20	4 (20.00)	16 (80.00)	**4.44 (1.16–16.95)**
**Finger pain:** 249	23 (9.24)	226 (90.76)	1.55 (0.93–2.57)
No Finger pain: 7241	353 (4.88)	6888 (95.12)	Ref
Mild Finger pain: 146	10 (6.85)	136 (93.15)	1.14 (0.55–2.36)
Moderate Finger pain: 89	12 (13.48)	77 (86.52)	**2.40 (1.16–4.96)**
Severe Finger pain: 14	1 (7.14)	13 (92.86)	1.09 (0.13–9.25)
**Chest pain:** 53	7 (13.21)	46 (86.79)	2.16 (0.89–5.27)
No Chest pain: 7437	369 (4.96)	7068 (95.04)	Ref
Mild Chest pain: 33	3 (9.09)	30 (90.91)	1.01 (0.27–3.74)
Moderate Chest pain: 17	4 (23.53)	13 (76.47)	**8.43 (2.34–30.37)**
Severe Chest pain: 3	0 (0)	3 (100)	NA
**Stomachache:** 29	3 (10.34)	26 (89.66)	2.08 (0.54–8.04)
No Stomachache: 7461	373 (5.00)	7088 (95.00)	Ref
Mild Stomachache: 20	2 (10.00)	18 (90.00)	2.24 (0.40–12.64)
Moderate Stomachache: 8	1 (12.50)	7 (87.50)	1.92 (0.27–17.03)
Severe Stomachache: 1	0 (0)	1 (100)	NA
**Back pain:** 2694	197 (7.31)	2497 (92.69)	**1.36 (1.07–1.72)**
No Back pain: 4796	179 (3.73)	4617 (96.27)	Ref
Mild Back pain: 1620	67 (4.14)	1553 (95.86)	0.84 (0.62–1.16)
Moderate Back pain: 878	101 (11.50)	777 (88.50)	**1.98 (1.47–2.65)**
Severe Back pain: 196	29 (14.80)	167 (85.20)	**2.45 (1.51–3.96)**
**Hip pain:** 264	43 (16.29)	221 (83.71)	**2.66 (1.80–3.93)**
No Hip pain: 7226	333 (4.61)	6893 (95.39)	Ref
Mild Hip pain: 123	11 (8.94)	112 (91.06)	1.34 (0.66–2.72)
Moderate Hip pain: 109	21 (19.27)	88 (80.73)	**2.98 (1.72–5.16)**
Severe Hip pain: 32	11 (34.38)	21 (65.63)	**7.85 (3.38–18.18)**
**Leg pain:** 2185	211 (9.66)	1974 (90.34)	**1.96 (1.54–2.48)**
No Leg pain: 5305	165 (3.11)	5140 (96.89)	Ref
Mild Leg pain: 1257	75 (5.97)	1182 (94.03)	1.22 (0.89–1.66)
Moderate Leg pain: 744	112 (15.05)	632 (84.95)	**3.14 (2.36–4.20)**
Severe Leg pain: 184	24 (13.04)	160 (86.96)	**2.37 (1.42–3.94)**
**Knee pain:** 2524	194 (7.69)	2330 (92.31)	1.16 (0.91–1.47)
No Knee pain: 4966	182 (3.66)	4784 (96.34)	Ref
Mild Knee pain: 1372	56 (4.08)	1316 (95.92)	0.66 (0.47–0.92)
Moderate Knee pain: 921	110 (11.94)	811 (88.06)	**1.87 (1.41–2.49)**
Severe Knee pain: 231	28 (12.12)	203 (87.88)	1.40 (0.85–2.31)
**Ankle pain:** 213	32 (15.02)	181 (84.98)	**2.36 (1.48–3.76)**
No Ankle pain: 7277	344 (4.73)	6933 (95.27)	Ref
Mild Ankle pain: 85	7 (8.24)	78 (91.76)	1.13 (0.43–2.99)
Moderate Ankle pain: 105	19 (18.10)	86 (81.90)	**2.93 (1.61–5.34)**
Severe Ankle pain: 23	6 (26.09)	17 (73.91)	**4.30 (1.39–13.34)**
**Toe pain:** 67	10 (14.93)	57 (85.07)	1.84 (0.80–4.23)
No Toe pain: 7423	366 (4.93)	7057 (95.07)	Ref
Mild Toe pain: 39	6 (15.38)	33 (84.62)	2.45 (0.87–6.90)
Moderate Toe pain: 20	4 (20.00)	16 (80.00)	2.51 (0.56–11.20)
Severe Toe pain: 8	0 (0)	8 (100.00)	NA
**Multisite pain**	**n (%)**		**OR (CI)**
Multisite pain (yes/no)	3070 (41)		**1.72 (1.34–2.21)**
No pain	2869 (38)		Ref
Pain at 1–6 site	4552 (61)		**1.49 (1.11–2.01)**
Pain at 7–12 site	69 (0.92)		**7.16 (3.60–14.26)**

All ORs adjusted for age, sex, and BMI; Multisite pain: pain at more than one site; ADL: Activities of daily living; BMI: Body mass index; OR: Odds ratio; CI: Confidence interval. Bold represent statistically significant.

## Data Availability

The datasets generated during and/or analyzed during the study are available from the corresponding author on request.
